# Quantitative ultrasound (QUS) in the evaluation of liver steatosis: data reliability in different respiratory phases and body positions

**DOI:** 10.1007/s11547-024-01786-y

**Published:** 2024-03-21

**Authors:** Aldo Rocca, Klara Komici, Maria Chiara Brunese, Giulia Pacella, Pasquale Avella, Chiara Di Benedetto, Corrado Caiazzo, Marcello Zappia, Luca Brunese, Gianfranco Vallone

**Affiliations:** 1 Department of Medicine and Health Sciences “Vincenzo Tiberio”, University of Molise, 86100 Campobasso, Italy; 2 Department of General Surgery, Center for Hepatobiliary and Pancreatic Surgery, Pineta Grande Hospital, Castel Volturno, CE Italy

**Keywords:** Artificial intelligence, Formal Methods, Pancreatitis, Radiomics

## Abstract

Liver steatosis is the most common chronic liver disease and affects 10–24% of the general population. As the grade of disease can range from fat infiltration to steatohepatitis and cirrhosis, an early diagnosis is needed to set the most appropriate therapy. Innovative noninvasive radiological techniques have been developed through MRI and US. MRI-PDFF is the reference standard, but it is not so widely diffused due to its cost. For this reason, ultrasound tools have been validated to study liver parenchyma. The qualitative assessment of the brightness of liver parenchyma has now been supported by quantitative values of attenuation and scattering to make the analysis objective and reproducible. We aim to demonstrate the reliability of quantitative ultrasound in assessing liver fat and to confirm the inter-operator reliability in different respiratory phases. We enrolled 45 patients examined during normal breathing at rest, peak inspiration, peak expiration, and semi-sitting position. The highest inter-operator agreement in both attenuation and scattering parameters was achieved at peak inspiration and peak expiration, followed by semi-sitting position. In conclusion, this technology also allows to monitor uncompliant patients, as it grants high reliability and reproducibility in different body position and respiratory phases.

## Introduction

The most common chronic liver disease is liver steatosis or fatty liver, which affects 10–24% of the general population [1]. Non-alcoholic fatty liver disease (NAFLD) is a chronic disease related not only to alcohol consumption but also to diabetes, hyperlipidemia, toxins, drugs, or genetic diseases [2, 3].

NAFLD is diffusing in the general population in two steps, the first step consists of metabolic syndrome, where insulin resistance induces liver parenchyma to stock fat developing liver steatosis [4, 5].

The second step provides the progression of liver steatosis and steatohepatitis (NASH), characterized by inflammation and chronic damage that may evolve into liver fibrosis and end-stage liver disease [6]. Late or delayed diagnosis without any lifestyle change may increase the risk of liver fibrosis or cirrhosis in the general population with consequent costs on the healthcare system, but even in patients after liver transplantation [7–10].

Liver biopsy has the limit of being an invasive procedure and it allows the examination of only a selected parenchyma, but it has been abandoned in clinical practice [11–13].

To overcome those limits innovative noninvasive radiological techniques have been developed through MRI and US [14–17].

Magnetic resonance imaging proton density fat fraction (MRI-PDFF) enables accurate, repeatable, and reproducible quantitative assessment of liver fat over the entire liver parenchyma, achieving high accuracy and sensitivity [18]. The diagnostic power of MRI-PDFF allows the detection even of the 5% of microscopic fat, so it has a higher sensitivity to detect early, but fundamental changes in liver fat content than liver biopsies [19, 20].

The major concern about the wide diffusion of MRI-PDFF is represented by its costs, limited diffusion, and patient compliance [21–23]. Besides standard protocols, radiomics has already been proposed to be a useful tool in the management of several pathologies [24–26].

More in detail, most recent studies are also proposing machine learning-based models to analyze liver parenchyma, but there are still few studies validated in clinical practice [15, 27, 28].

Considering the huge diffusion and reproducibility of the liver US, ultrasound software has been enriched by tools dedicated to hepatic fat quantification [29].

Despite it is well known that a high percentage of fat determines the brightness of liver parenchyma on US images, we have to underline that this is related to the scatter and to the attenuation of the ultrasound wave due to the amount of fats [30, 31].

In particular, B-Mode ultrasound allows to assess the grade of liver steatosis through: the evaluation of echogenicity of the liver compared to the renal cortex. Furthermore, the right liver attenuation with diaphragm visualization and the visualization of intra-hepatic vessels are commonly used in clinical practice [32].

The qualitative assessment of the morphology or the brightness of liver parenchyma has now been supported by quantitative values obtained from tissue microstructure characterization [33] through quantitative ultrasound (QUS) techniques.

There is only a few evidence about the inter-operator reliability of fat quantification tools in clinical practice [34]. There is also a lack of evidence of its reliability in selected categories of patients, in different body positions and respiratory phases [35].

So this study aims to demonstrate the reliability of quantitative ultrasound (QUS) in assessing liver fat volume measurements, to confirm the inter-operator reliability in the respiratory phases, but even in different body positions, in order to follow up uncompliant patients.

## Tissue attenuation imaging (TAI, Samsung Medison)

Tissue attenuation is due to the energy loss of an ultrasound wave when it passes through a tissue. Attenuation depends on tissue features and wave frequency. When the percentage of liver fat is higher also the attenuation increases [36].

Attenuation coefficient (AC) has been calculated with several methods proposed by different vendors [37–39]. AC showed high reliability to detect liver fat, and to estimate the grade of liver steatosis, compared to liver biopsy and MRI-PDFF as reference standard [40].

In our study, AC is calculated by a parameter, the tissue attenuation imaging (TAI, Samsung Medison), that indicates the slope of the ultrasound central frequency downshift along the depth.

## Tissue scatter distribution imaging (TSI, Samsung Medison)

Backscattering refers to the ultrasound energy reflected from a tissue, and it is represented by echogenicity or brightness. In particular, liver brightness means the backscattering has increased. The scattering of ultrasound also creates images with speckle patterns. The different patterns have been described by a statistical distribution. In particular, the Nakagami distribution correlates backscattering to the percentage of liver fat. In our work, Nakagami distribution is calculated by a parameter, tissue scatter distribution imaging (TSI, Samsung Medison), which calculates the concentration and the distribution of the ultrasound scatterers [41, 42].

TSI has been validated through a comparison with liver biopsy and MRI-PDFF as reference standards.

## Materials and methods

This is a retrospective study conducted on a prospectively collected database study conducted at the University of Molise between November 2022 and April 2023. The patients were admitted to an abdominal ultrasound examination for other reasons, at the University of Molise, Campobasso, Italy.

All patients signed an informed consent to publish their anonymous clinical data.

Inclusion criteria:

- > 18 years old.
- No history of chronic liver disease.
- No habitual alcohol consumption.

Exclusion criteria:

- < 18 years old.
- Chronic liver disease or alcohol addiction.
- Lack of compliance.

We studied echogenicity and composition of liver parenchyma.

For each patient beyond the US exam, we provided a dataset of clinical data: gender, age, body mass index (BMI), complete blood count, bilirubin and alanine aminotransferase (ALT), and aspartate aminotransferase (AST) levels.

Patients were divided into three subgroups according to their body mass index: Subgroup 1 includes normal-weight patients with 18.5 < BMI < 24.99 kg/m^2^; subgroup 2 includes overweight patients with 25 < BMI < 29.99 kg/m^2^; and subgroup 3 includes obese patients with BMI > 30 kg/m^2^.

The ultrasound exam was performed on the right lobe with Samsung RS85 Prestige, with a single convex transducer 1–7 MHz convex transducer (CA1-7S) and completed with quantitative ultrasound (QUS) imaging: tissue attenuation imaging (TAI) and tissue scatter distribution imaging (TSI).

TAI was recorded only after verification of an “R2 value” < 0.8 [43].

The examinations were performed by 2 expert radiologists. A total of 10 measurements were recorded in different liver segments, in particular V, VI, VIII, and VII segments.

We included in the study the highest value of TAI and TSI found by each physician.

For each patient, a mean of 10 measurements was recorded using four different methods:

- Method 1: normal breathing at rest.
- Method 2: peak inspiration.
- Method 3: end-expiration.
- Method 4: semi-sitting position.

Patients were asked to inhale, hold their breath, exhale, or breathe quietly. Then, patients were asked to move to a semi-seating position.

Operators 1 and 2 conducted the US with the same machine settings.

**Fig. 1:**
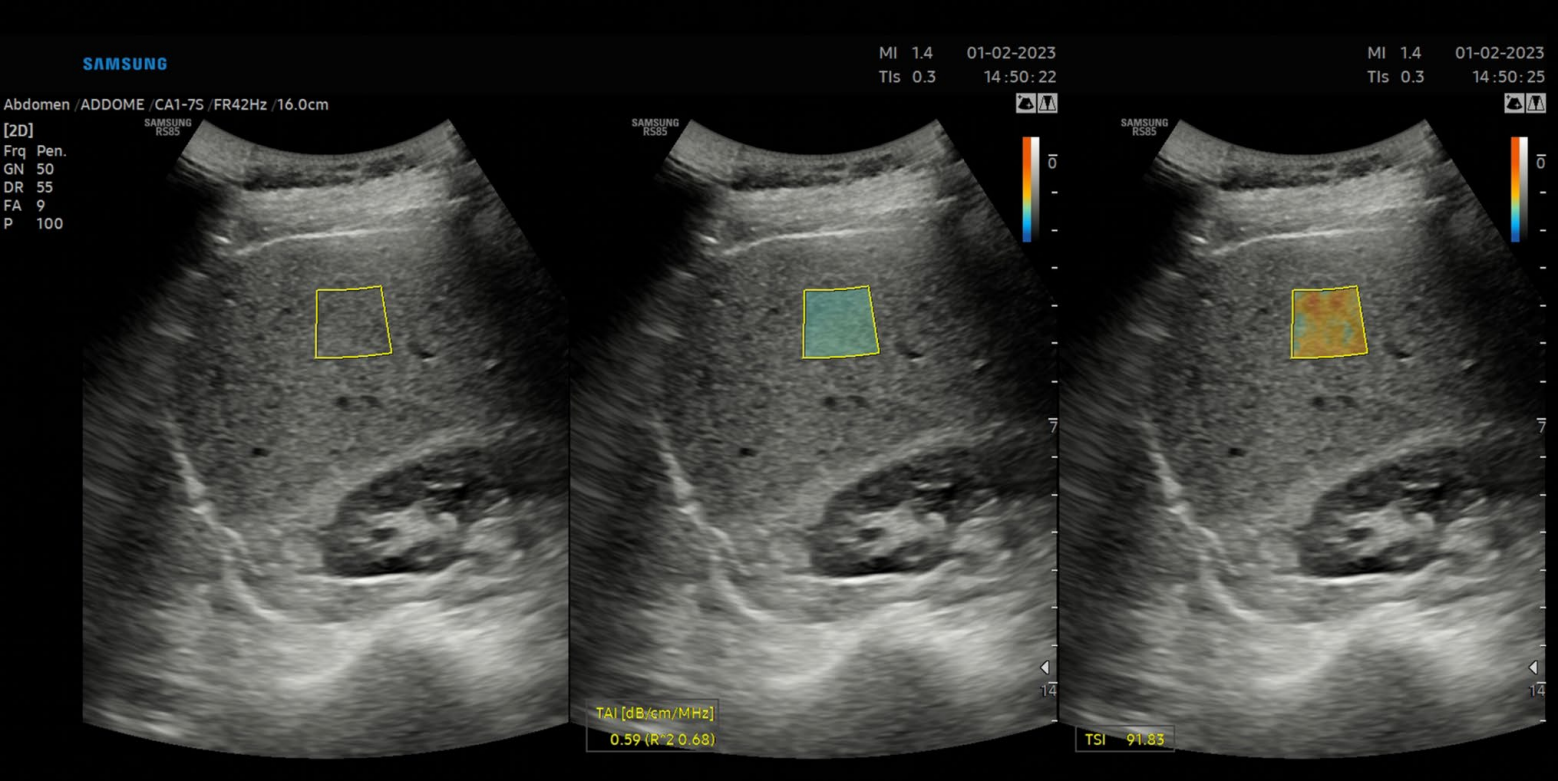
TAI and TSI measurement in a patient with BMI < 25 kg/m^2^. QUS shows no evidence of liver steatosis (Grade 0)

**Fig. 2:**
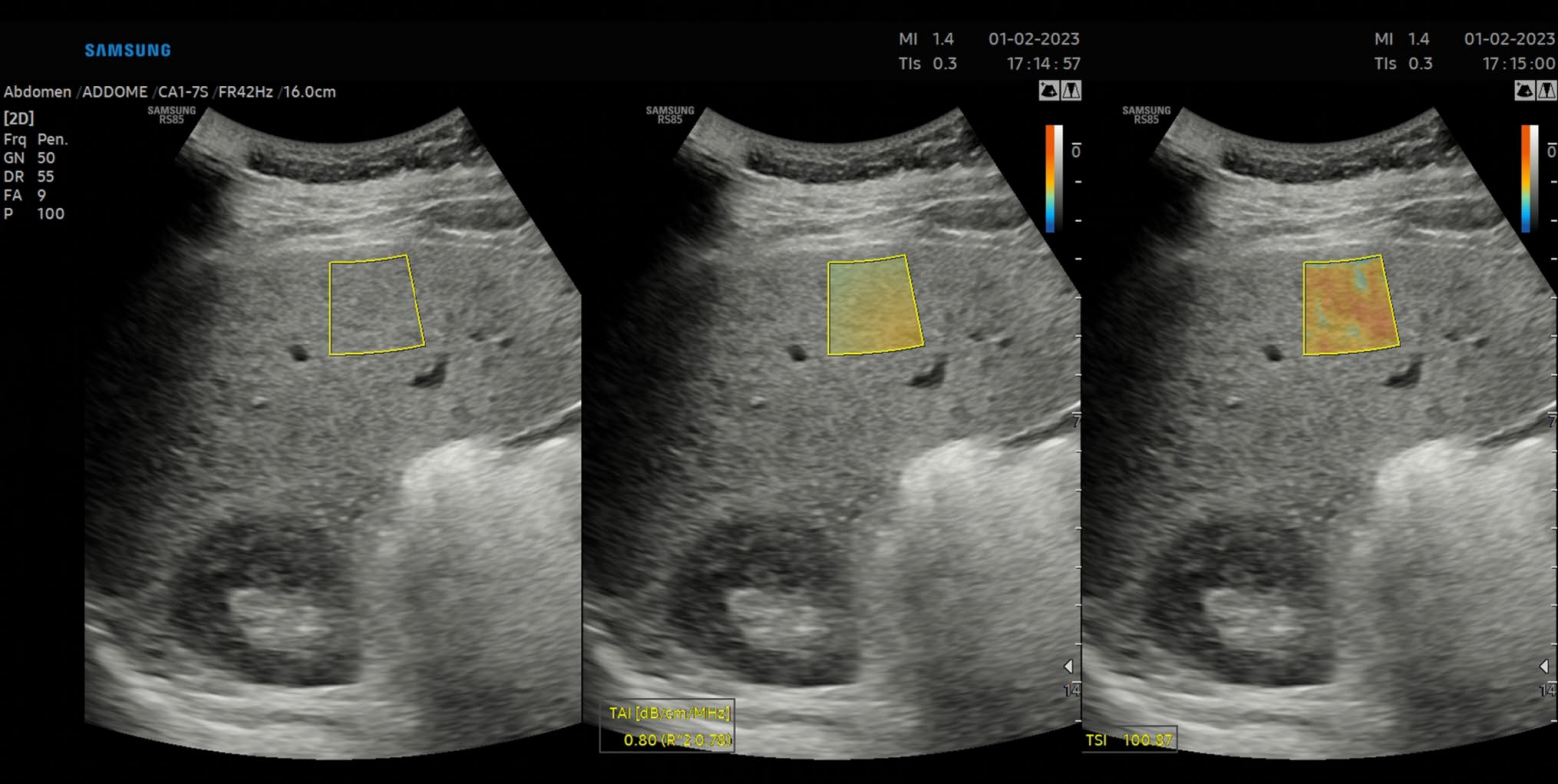
TAI and TSI measurement in a patient with BMI 25–30 kg/m^2^. QUS shows evidence of mild liver steatosis (Grade 2)

**Fig. 3:**
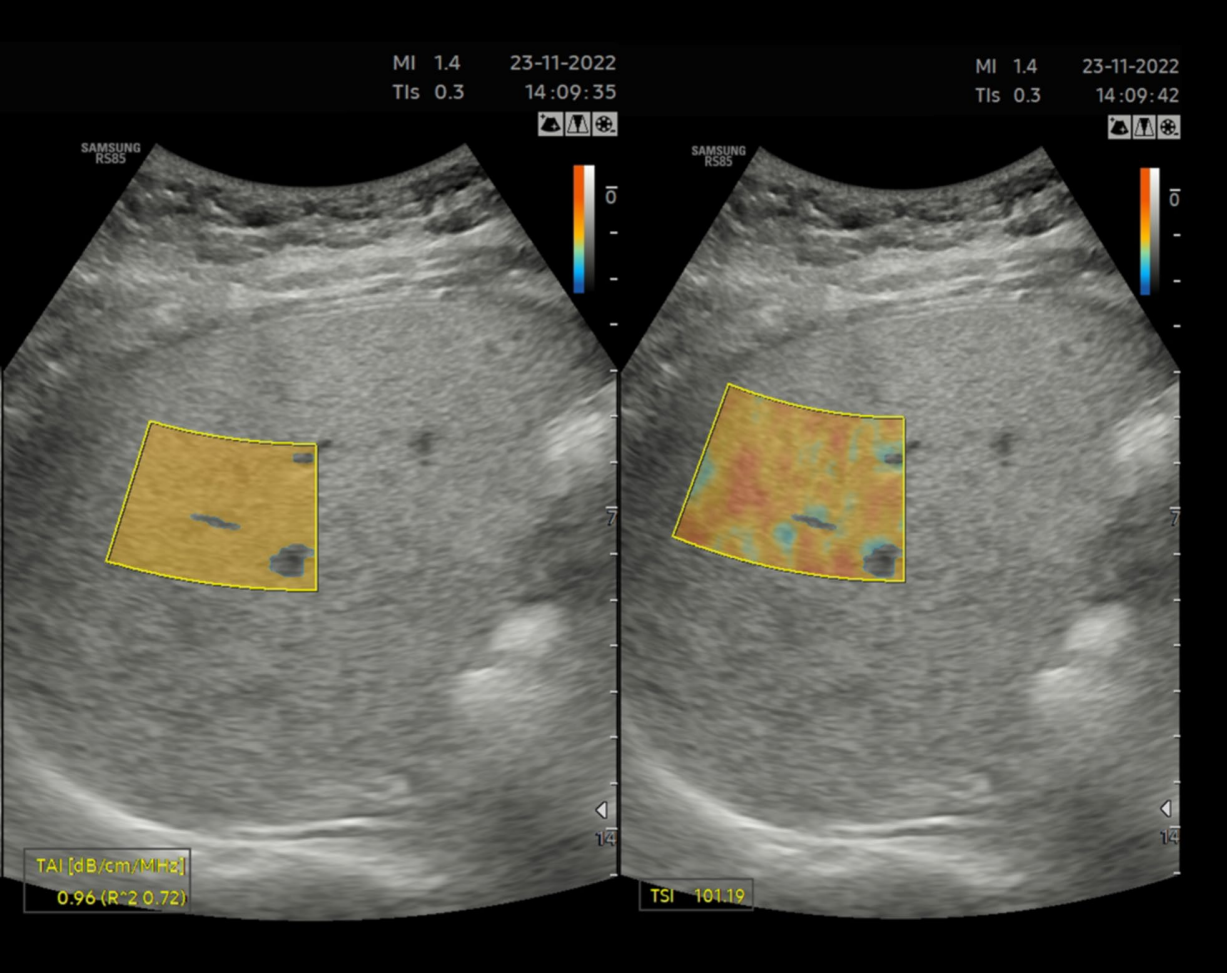
TAI and TSI measurement in a patient with BMI > 30 kg/m^2^. QUS shows evidence of severe steatosis (Grade 3)

Patient subgroups during examination are reported in Figs. 1, 2, and 3.

### Standard of reference

Table 1 reported the reference standard selected following Sendur et al. [41]

**Table 1:** Reference standard for steatosis grade quantification

Steatosis	MRI-PDFF value	TAI	TSI
Grade 1	MRI ≥ 5%	0.75	92.44
Grade 2	MRI ≥ 16.3%	0.86	96.64
Grade 3	MRI ≥ 21.7%	0.96	99.45

### Statistical analysis

Cohen's Kappa values were calculated to identify rates of inter-rater agreement between two different radiologists. Data were expressed as agreement in percentage, Cohen's Kappa value, standard error, and Z. The measure of the agreement below 0.0 means poor agreement, 0.00–0.20 slight agreement, 0.21–0.40 fair agreement, 0.41–0.60 moderate agreement, 0.61–0.80 substantial agreement, and > 0.80 almost perfect agreement [44, 45]. Considering that measurements were performed in different methods as normal breathing, peak inspiration, end-expiration, and semi-seating position, one-way ANOVA with Bonferroni correction was performed separately for both experts. Statistical significance was at *p* ≤ 0.05. Statistical analyses were performed with STATA SE 16.1 StataCorp LLC software.

## Results

We retrospectively collected 45 patients and divided them into three subgroups: 15 normal-weight patients, 15 overweight patients, and 15 obese patients. The mean age of the patients was 36.7 ± 1.89 [24–65 CI].

Most of the patients were male (27/45, 60%). In particular, most of the obese patients were male (10/15, 75%).

The mean values of TAI and TSI recorded by the operators during normal breath were 0.718 ± 0.026 (operator 1), 0.755 ± 0.236 (operator 2) and 92,654 ± 1465 (operator 1) 92579 ± 2549 (operator 2). TAI and TSI values are both expressions of a population with a mean grade of steatosis 1. Inter-operators agreement in this phase was low, 15.56% for TAI and 2.22% for TSI.

During forced inspiration, the mean values of TAI and TSI recorded by the operators were 0.728 ± 0.023 (operator 1), 0.741 ± 0.0219 (operator 2) and 93.67 ± 1.809 (operator 1) 94.33 ± 1.84 (operator 2). Both the TAI and TSI values are coherent and belong to steatosis grade 1. In this respiratory phase, the inter-operator agreement was higher both for TAI and TSI measurement, 48.89% and 37.78%, respectively.

During peak expiration, the mean values of TAI and TSI recorded by the operators were 0.736 ± 0.024 (operator 1), 0.732 ± 0.029 (operator 2), and 92,658 ± 1605 (operator 1), 92,571 ± 1608 (operator 2). Also during this respiratory phase, TAI and TSI values are coherent and belong to steatosis grade 1. Inter-operator agreement was the highest achieved, both for TAI and TSI measurement, 48.89% and 48.89%, respectively.

In the semi-sitting position, in uncompliant patients, the mean values of TAI and TSI recorded by the operators were 0.701 ± 0.02 (operator 1), 0.715 ± 0.021 (operator 2), and 92,098 ± 1316 (operator 1), 94,277 ± 1457 (operator 2). Inter-operator agreement as medium–high, 33.33% both for TAI and TSI values.

Inter-operator agreement calculated with K-Cohen test showed the lowest K values in the measurement of TAI and TSI during quiet breath (K = 0.137 and K = 0.0115, respectively).

ANOVA test showed a significant statistical difference among operators only in the TSI measurements; therefore, quiet breath strongly influenced TSI value rather than TAI.

Results of statistical analysis are summarized in Tables 2 and 3.

**Table 2:** The means of TAI and TSI values taken by each operator and the agreement between the measurements (K Cohen test)

	Total population					
	Age 36,7 ± 1,89					
	1	2	Agreement (%)	Kappa	Standard error	Z
TAI (rt)	0.718 ± 0.026	0.741 ± 0.0219	15.56	0.137	0.0247	5.29
TSI (rt)	92.654 ± 1.465	92.579 ± 2.549	2.22	0.0115	0.0149	0.77
TAI (insp)	0.728 ± 0.023	0.755 ± 0.236	48.89	0.4687	0.0287	16.33
TSI (insp)	93.67 ± 1.809	94.33 ± 1.84	37.78	0.3585	0.0244	14.69
TAI (esp)	0.736 ± 0.024	0.732 ± 0.029	48.89	0.4626	0.0319	14.48
TSI (esp)	92.658 ± 1.605	92.571 ± 1.608	48.89	0.4736	0.0248	19.13
TAI (semi)	0.701 ± 0.02	0.715 ± 0.021	33.33	0.3056	0.0288	10.60
TSI (semi)	92.098 ± 1.316	94.277 ± 1.457	33.33	0.3151	0.0235	13.40

**Table 3:** The TAI and TSI measurements in each method (ANOVA test)

	Method 1 N = 45	Method 2 N = 45	Method 3 N = 45	Method 4 N = 45	*P* value
TAI1	0.7184 ± 0.1798	0.7288 ± 0.1597	0.736 ± 0.1668	0.7015 ± 0.1358	0.316
TSI1	92.6546 ± 9.8317	93.670 ± 12.1415	92.6586 ± 10.7699	92.0986 ± 8.8323	0.189
TAI2	0.7406 ± 0.1474	0.7551 ± 0.1584	0.7322 ± 0.1967	0.7155 ± 0.1446	0.136
TSI2	92.5797 ± 17.1057	94.3384 ± 12.3456	92.5717 ± 10.7921	94.2773 ± 9.7743	0.001

## Discussion

This study aimed to evaluate the impact on TAI and TSI values of the breathing cycle, chest movement, and body position, to validate the reliability of QUS. This validation allows operators to monitor even hospitalized or uncompliant patients in the most appropriate position or respiratory phase to overcome the limitations due to several artifacts. As for all new technologies the reliability and reproducibility of QUS are not completely tested.

After a statistical analysis, the ultrasound quantification of liver fat is confirmed to be reliable even in normal-weight patients, even in overweight patients, and even obese patients. The evaluation of attenuation and scattering achieved a high agreement among the operators, especially during the peak inspiration and peak expiration, but also a satisfying agreement in the semi-sitting position. The study conducted by Sendur et al. reported that inspiration and expiration do not significantly influence the results in patients with BMI > 25 kg/m^2^, while a significant difference in the attenuation coefficient in the BMI < 25 kg/m^2^ subgroup was found. Our study confirmed the reliability among the respiratory phases in overweight patients (BMI > 25 kg/m^2^), but also among the operators. In addition, there was not a significant difference in patients with BMI < 25 kg/m^2^ among the operators, among different respiratory phases. Also, in the BMI < 25 kg/m^2^ subgroup, there was a stronger agreement at peak inspiration and expiration, in spite of quiet breath. Concerning the different methods evaluated in our study, TAI measurement did not show any statistically significant difference among the respiratory phases, while TSI did, due to the higher variability in the quiet breath. This was also probably due to a higher sensitivity of the method to the thorax movement.

Affordability, portability, and wide availability are some of the many advantages of ultrasound in clinical practice, in comparison with other imaging techniques. Therefore, US tools could be efficiently used to diagnose and follow-up liver steatosis.

Comparing our data with the reference standard reported by Sendur et al., in our dataset there was neither underestimation nor overestimation of steatosis grade attributed to the patients among different methods [34, 46].

Anyway in this study, we focused on demonstrating the reliability of TAI and TSI measurements inter-operators in a stratified population composed of normal, overweight, and obese patients.

The main limitation of our study is the unavailability of an MRI-PDFF to compare the results. To overcome this limit we introduced a control group of normal-weight patients not affected by liver steatosis. TAI and TSI measurements, in fact, gradually increased from the normal-weight group to obese group.

The importance of detecting liver steatosis is already assessed as it affects 90% of obese patients [47].

Because the grade of injury can range from fat infiltration to cirrhosis, early therapy must be set [48, 49] and monitored to lose weight, rather than muscle mass [50–52].

Nowadays, the most effective treatments are represented by bariatric surgery in young patients [53], to avoid the development of metabolic syndrome and liver failure. It should be underlined, the high risk of liver steatosis also after liver transplants [54, 55], so QUS may represent a safe and efficient tool to monitor the results of bariatric surgery or the health of the liver graft.

Even if bariatric surgery might be a challenging surgical procedure and it might be considered an invasive treatment, especially for young people, the advent of minimally invasive surgery has changed surgical scenarios allowing faster recovery, lower blood loss, and lower risk of major complications [56–64].

Thanks to the low risk of complications, several studies are now introducing a combined treatment with both bariatric surgery before liver transplantation, as NAFLD is a metabolic condition that may persist in damaging the graft [65–67].

The combined treatment can be helpful in adult or elderly patients, even if a consensual physical performance is needed and further studies are needed to standardize the procedure [68, 69].

In these groups of patients who have undergone bariatric surgery or liver transplantation, the importance of follow-up to monitor liver fat is outstanding and there is the need to quantify the percentage of liver fat to avoid sub-optimal treatments [70].

In addition, as liver fibrosis can benefit weight loss, bariatric surgery is starting to be considered also in compensated patients [71].

Future investigation will focus on the implementation and validation of the shear wave parameter to evaluate and monitor liver fibrosis.

Future studies will concern the evaluation of attenuation and scattering in a prospective cohort of patients undergoing bariatric surgery and weight loss.
